# Recipient-Biased Competition for an Intracellularly Generated Cross-Fed Nutrient Is Required for Coexistence of Microbial Mutualists

**DOI:** 10.1128/mBio.01620-17

**Published:** 2017-11-28

**Authors:** Alexandra L. McCully, Breah LaSarre, James B. McKinlay

**Affiliations:** Department of Biology, Indiana University, Bloomington, Indiana, USA; Georgia Institute of Technology; University of Rhode Island

**Keywords:** cross-feeding, coculture, fermentation, hydrogen, microbial communities, mutualism, nitrogen fixation, purple bacteria, synthetic ecology

## Abstract

Many mutualistic microbial relationships are based on nutrient cross-feeding. Traditionally, cross-feeding is viewed as being unidirectional, from the producer to the recipient. This is likely true when a producer’s waste, such as a fermentation product, has value only for a recipient. However, in some cases the cross-fed nutrient holds value for both the producer and the recipient. In such cases, there is potential for nutrient reacquisition by producer cells in a population, leading to competition against recipients. Here, we investigated the consequences of interpartner competition for cross-fed nutrients on mutualism dynamics by using an anaerobic coculture pairing fermentative *Escherichia coli* and phototrophic *Rhodopseudomonas palustris*. In this coculture, *E. coli* excretes waste organic acids that provide a carbon source for *R. palustris*. In return, *R. palustris* cross-feeds *E. coli* ammonium (NH_4_^+^), a compound that both species value. To explore the potential for interpartner competition, we first used a kinetic model to simulate cocultures with varied affinities for NH_4_^+^ in each species. The model predicted that interpartner competition for NH_4_^+^ could profoundly impact population dynamics. We then experimentally tested the predictions by culturing mutants lacking NH_4_^+^ transporters in both NH_4_^+^ competition assays and mutualistic cocultures. Both theoretical and experimental results indicated that the recipient must have a competitive advantage in acquiring cross-fed NH_4_^+^ to sustain the mutualism. This recipient-biased competitive advantage is predicted to be crucial, particularly when the communally valuable nutrient is generated intracellularly. Thus, the very metabolites that form the basis for mutualistic cross-feeding can also be subject to competition between mutualistic partners.

## IMPORTANCE

Mutualistic relationships, particularly those based on nutrient cross-feeding, promote stability of diverse ecosystems and drive global biogeochemical cycles. Cross-fed nutrients within these systems can be either waste products valued by only one partner or nutrients valued by both partners. Here, we explored how interpartner competition for a communally valuable cross-fed nutrient impacts mutualism dynamics. We discovered that mutualism stability necessitates that the recipient have a competitive advantage against the producer in obtaining the cross-fed nutrient, provided that the nutrient is generated intracellularly. We propose that the requirement for recipient-biased competition is a general rule for mutualistic coexistence based on the transfer of intracellularly generated, communally valuable resources.

## INTRODUCTION

Mutualisms, or mutually beneficial relationships between organisms, are ubiquitous and play important roles in diverse ecosystems (1). Mutualistic cross-feeding of resources between microbes can have broad impacts, ranging from influencing host health (2, 3) to driving global biogeochemical cycles (4–7). Cross-fed metabolites are often regarded as nutrients due to the value they provide to a dependent partner, the recipient. However, for the partner producing the nutrient, the producer, a cross-fed nutrient’s value can vary. On one extreme, the cross-fed metabolite is valued by the recipient but not the producer, as is the case for fermentative waste products (8–11). In other cases, a cross-fed metabolite holds value for both the recipient and the producer, as is the case for vitamin B_12_ (7, 12, 13) and ammonium (NH_4_^+^) (14, 15). Such communally valuable cross-fed nutrients are subject to partial privatization (16), wherein the producer has mechanisms to retain a portion of the nutrient pool for itself. While most mutualism cross-feeding studies only consider unidirectional metabolite transfer from producer to recipient, we hypothesized that partially privatized cross-fed resources could be subject to competition between partner populations. Such competition from partial privatization mechanisms seems likely, considering that competition for exogenous limiting resources is known to affect mutualism stability (9, 17–20). Similarly, others have shown that adding an exogenous source of a cross-fed nutrient can shift relationships between microbial partners from being mutualistic to competitive (21).

**FIG 1:**
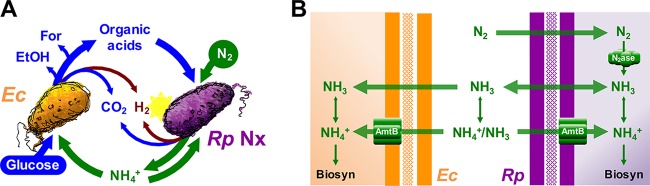
An obligate bacterial mutualism based on cross-feeding of essential nutrients. (A) *Escherichia coli* (*Ec*) anaerobically ferments glucose into excreted organic acids that *Rhodopseudomonas palustris* Nx (*Rp* Nx) can consume (acetate, lactate, and succinate) and other products that *R. palustris* cannot consume (formate [For] and ethanol [EtOH]). *R. palustris* Nx grows photoheterotrophically, wherein organic compounds are used for carbon and electrons and light is used for energy. In return, *R. palustris* Nx constitutively fixes N_2_ gas and excretes NH_4_^+^, supplying *E. coli* with essential nitrogen. (B) NH_4_^+^ can be passively lost from cells as NH_3_. Both species have high-affinity NH_4_^+^ transporters (AmtB) that facilitate NH_4_^+^ uptake. NH_4_^+^ is the predominant form at neutral pH, as indicated by the enlarged arrowheads of the double-sided arrows.

One example of cross-feeding that could involve competition between mutualistic partners is NH_4_^+^ excretion by N_2_-fixing bacteria (Fig. 1A), hereon called N_2_ fixers (14, 15). During N_2_ fixation, the enzyme nitrogenase converts N_2_ gas into two NH_3_ molecules (22). In an aqueous environment, NH_3_ is in equilibrium with NH_4_^+^. At neutral pH, NH_4_^+^ is the predominant form, but small amounts of NH_3_ can potentially leave the cell by passive diffusion across the membrane; this passive diffusion is referred to here as NH_4_^+^ excretion (23) (Fig. 1B). This inherent “leakiness” for NH_3_ likely fosters NH_4_^+^ cross-feeding, as extracellular NH_3_ is available to neighboring microbes. Importantly, these neighbors can include clonal N_2_ fixers, as NH_3_/NH_4_^+^ is a preferred nitrogen source for most microbes. At concentrations above 20 μM, extracellular NH_3_ can be acquired by passive diffusion; below 20 μM, NH_4_^+^ is specifically bound and transported as NH_3_ by AmtB transporters (Fig. 1B) (24). AmtB-like transporters are conserved throughout all domains of life (25). There is growing evidence that AmtB is used by N_2_ fixers to recapture excreted NH_3_ lost by passive diffusion, as ΔAmtB mutants accumulate NH_4_^+^ in culture supernatants, whereas wild-type strains do not (26–28). Thus, during NH_4_^+^ cross-feeding, AmtB likely facilitates both NH_4_^+^ acquisition by a recipient partner and recapture of NH_4_^+^ by the N_2_ fixer.

Assessment of the effects of interpartner competition for a cross-fed nutrient would require a level of experimental control not possible in most natural settings. However, synthetic microbial communities, or cocultures, are well-suited to address such questions (29–31). We previously developed a bacterial coculture that features cross-feeding of waste products (organic acids) from *Escherichia coli* and a communally valuable nutrient (NH_4_^+^) from *Rhodopseudomonas palustris* Nx (Fig. 1A) (28). We demonstrated that this coculture supports stable coexistence and reproducible growth and metabolic trends when started from a wide range of starting species ratios, including single colonies (28). Here, using both a kinetic model and genetic manipulation to alter the affinity of each species in the coculture for NH_4_^+^, we demonstrate that interpartner competition for excreted NH_4_^+^ plays a direct role in maintaining coexistence. Specifically, insufficient competition by *E. coli* for NH_4_^+^ resulted in a collapse of the mutualism. Mutualism collapse could be delayed or potentially avoided through higher NH_4_^+^ excretion by *R. palustris* or increased *E. coli* population size. Our results suggest that for obligate mutualisms based on an intracellularly generated cross-fed nutrient, competition for that nutrient must be biased in favor of the recipient to avoid mutualism collapse and the potential extinction of both species.

## RESULTS

### Competition for cross-fed NH_4_^+^ is predicted to shape mutualism population dynamics.

Within our coculture (Fig. 1A), *E. coli* ferments sugars into waste organic acids, providing essential carbon and electrons to *R. palustris* Nx. *R. palustris* Nx converts N_2_ into NH_4_^+^ and is genetically engineered to excrete low micromolar amounts of NH_4_^+^, providing essential nitrogen for *E. coli* (28). The *R. palustris* parent strain does not support coculture growth with *E. coli* due to insufficient NH_4_^+^ excretion (28). NH_4_^+^ excretion by *R. palustris* Nx is due to a 48-nucleotide internal deletion in the gene for the master transcriptional regulator of nitrogenase, *nifA*, which results in constitutive nitrogenase activity even in the presence of normally inhibitory NH_4_^+^ (32). In contrast to organic acids, which are only useful to *R. palustris*, NH_4_^+^ produced by *R. palustris* Nx is essential for the growth of both species; *R. palustris* uses some NH_4_^+^ that it converted from N_2_ for its own biosynthesis and excretes the rest, which serves as the nitrogen source for *E. coli*. However, *R. palustris* Nx can also take up extracellular NH_4_^+^ (32). Thus, we hypothesized that competition for excreted NH_4_^+^ between the *R. palustris* Nx producer population and the *E. coli* recipient population could influence mutualism dynamics.

**FIG 2:**
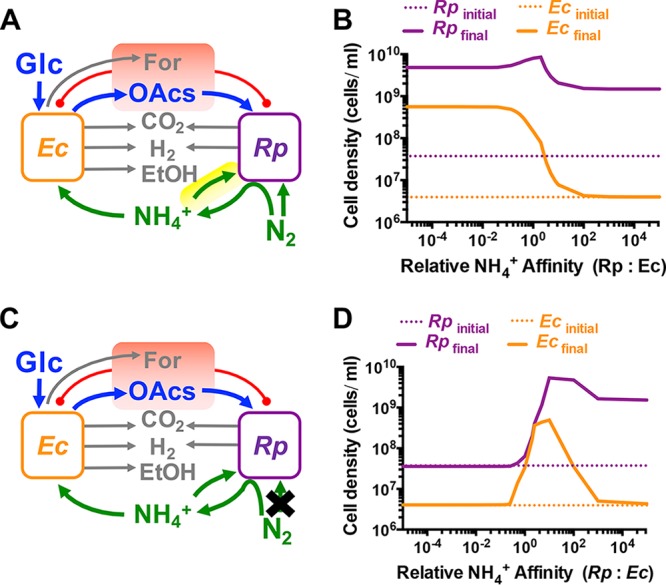
Simulations suggested that *E. coli* must have a competitive advantage for NH_4_^+^ acquisition relative to *R. palustris* to support mutualistic growth. (A) The default SyFFoN version, which enforces partial privatization of NH_4_^+^ by allowing *R. palustris* (*Rp*) to directly use N_2_. The yellow highlighted *R. palustris* NH_4_^+^ uptake arrow is new to the default SyFFoN version used here. Red highlighting indicates that both formate (For) and consumable organic acids (OAcs; succinate, lactate, and acetate) can inhibit growth and metabolism if they accumulate enough to acidify the medium. (B) Simulated population trends from the model in panel A. (C) Modified SyFFoN version where all NH_4_^+^ made from N_2_ is available to both species by removing the direct utilization of N_2_ by *R. palustris* (black X). See Text S1 and Table S2 for more details. (D) Simulated population trends from the model in panel C. (B and D) Final cell densities (solid lines) of *R. palustris* and *E. coli* after 300 h in simulated batch cultures for a range of relative NH_4_^+^ affinities. Starting cell densities (dashed lines) were based on a 1% dilution of cocultures containing 10% *E. coli*, as has been observed experimentally (28). Affinity is taken to be the inverse of *K_m_*. Therefore, relative NH_4_^+^ affinity values represent the *E. coli K*_*m*_ for NH_4_^+^ (*K*_*A*_) divided by that of *R. palustris* (*K*_AR_). For a ratio of 1, each species had a default *K*_*m*_ for NH_4_^+^ of 0.01 mM. To the left of 1, the *R. palustris K*_*m*_ value was raised. To the right of 1, the *E. coli K*_*m*_ value was raised. The peak and then decline in *R. palustris* cell density as its affinity for NH_4_^+^ increased is an artifact of the amount of organic acids that nongrowing *E. coli* cells excreted by 300 h, as the peak was not observed when more time was simulated (Fig. S2).

We first explored whether competition for cross-fed NH_4_^+^ could affect the mutualism by using SyFFoN, a mathematical model describing our coculture (28, 33). SyFFoN simulates population and metabolic dynamics in batch cocultures based on Monod equations with experimentally determined parameter values. Graphical details for individual functions and parameter value choices have been described elsewhere (33). As previous versions described NH_4_^+^ uptake kinetics only for *E. coli* (28, 33), we amended SyFFoN to include both an *R. palustris* NH_4_^+^ uptake affinity constant (*K*_*m*_) and a higher *R. palustris* maximum growth rate (μ_MAX_) when NH_4_^+^ is used (Fig. 2A; see also Table S1 and Text S1). Simulations from the amended model, SyFFoN v3, and the previous version, SyFFoN v2 (33), were comparable (Fig. S1). We then simulated batch cocultures, wherein the relative affinity for NH_4_^+^ varied between the two species by increasing the *K*_*m*_ value for NH_4_^+^ from the default value of 0.01 mM in either species (Fig. 2B). We did not decrease *K*_*m*_ values, because NH_4_^+^ transporters are regarded as high-affinity transporters (34), and therefore we assumed that a higher affinity was less likely physiologically. The model predicted that net growth of both species is achieved only when the *R. palustris* affinity for NH_4_^+^ is low relative to that of *E. coli* (*R. palustris*:*E. coli* affinity ratio, <1; herein affinity values are the inverse of *K_m_* values), as *E. coli* can acquire enough excreted NH_4_^+^ to be able to grow. In contrast, when the *R. palustris* affinity for NH_4_^+^ is high relative to that of *E. coli* (*R. palustris*:*E. coli* affinity ratio, >1), *E. coli* growth is no longer supported, because *E. coli* cannot compete for excreted NH_4_^+^. These trends are minimally impacted by the increase in the *R. palustris* growth rate when reacquiring NH_4_^+^ (Fig. S2). Changing the default *K*_*m*_ value (e.g., to 1 μM) affected the simulated cell density values but not the overall trends. Despite the lack of *E. coli* growth, high *R. palustris* cell densities were still predicted (Fig. 2B), due to persistent, low-level organic acid cross-feeding stemming from *E. coli* maintenance metabolism, which can support *R. palustris* growth even when *E. coli* is not growing (33). In contrast, NH_4_^+^ cross-feeding from *R. palustris* to *E. coli* functions solely in a growth-dependent manner, as the organic acids from *E. coli* serve both as the electron source for nitrogenase and the carbon source for *R. palustris* growth.

10.1128/mBio.01620-17.9 TABLE S1  Default and alternative parameter values used in SyFFoN v3 (unless stated otherwise). Download TABLE S1, DOCX file, 0.02 MB. Copyright © 2017 McCully et al. 2017 McCully et al. This content is distributed under the terms of the Creative Commons Attribution 4.0 International license.

10.1128/mBio.01620-17.1 TEXT S1  SyFFoN v3 equations and parameter descriptions. Download TEXT S1, DOCX file, 0.1 MB. Copyright © 2017 McCully et al. 2017 McCully et al. This content is distributed under the terms of the Creative Commons Attribution 4.0 International license.

**FIG 3:**
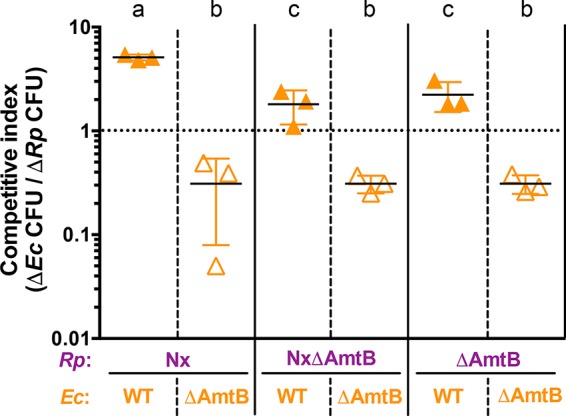
AmtB is important for competitive NH_4_^+^ acquisition. Competitive indexes for *E. coli* after 96 h in NH_4_^+^-limited competition assay cocultures. Cocultures were inoculated with *E. coli* and *R. palustris* at equivalent cell densities with excess carbon available for each species (25 mM glucose for *E. coli* and 20 mM sodium acetate for *R. palustris*). NH_4_^+^ was added to cocultures to a final concentration of 5 μM every hour for 96 h, a concentration at which AmtB is important for NH_4_^+^ uptake (24). The dotted line indicates a competitive index value of 1, where both species are equally competitive for NH_4_^+^. Filled triangles, WT *E. coli*; open triangles, *E. coli* ΔAmtB. Error bars indicate standard deviations (*n* = 3). Different letters indicate statistical differences between competitive index values (*P* < 0.05, determined by one-way analysis of variance with Tukey’s multiple-comparisons posttest).

10.1128/mBio.01620-17.2 FIG S1  SyFFoN v2 and v3 predict similar cell densities and product concentrations. Predictions from SyFFoN v2 and SyFFoN v3 and empirical data for final cell densities (A) and product yields (B to E) are shown. Batch cultures (300 h) were simulated. SyFFoN v3 includes the roles of relative NH_4_^+^ affinities, whereas SyFFoN v2 does not. R_A_ values for *R. palustris* NH_4_^+^ excretion were set to 0.15 fmol/cell and 0.5 fmol/cell for *R. palustris* strains Nx and Nx ΔAmtB, respectively. For SyFFoN v3, two simulations were performed using either equivalent NH_4_^+^ affinities or a slightly higher *E. coli* NH_4_^+^ affinity, based on the relative competitive affinities from Fig. 3 (*K*_A_, 0.01 mM; *K*_AR_, 0.05 mM). Symbols represent simulated values. Bars represent standard deviations from empirical data (*n* = 4). Simulated starting cell densities in panel A are indicated by dotted lines. Download FIG S1, TIF file, 0.4 MB. Copyright © 2017 McCully et al. 2017 McCully et al. This content is distributed under the terms of the Creative Commons Attribution 4.0 International license.

10.1128/mBio.01620-17.3 FIG S2  Simulation of longer (2,000 h) batch cultures and omitting the *R. palustris* growth rate boost on NH_4_^+^ do not change overall model predictions. (A) Simulation of 2,000-h cocultures instead of 300 h, as in Fig. 2B, allowed sufficient time for complete conversion of glucose into consumable organic acids by the smaller *E. coli* population and higher *R. palustris* final cell densities. (B) Omitting the boost in the *R. palustris* growth rate on NH_4_^+^ did not affect the model trends. To prevent the boosted growth rate, μRpMax2 was changed from 0.0152 h^−1^ (A) to 0 h^−1^ (B) for simulated 2,000-h batch cultures. (A, B) Initial cell densities are indicated by dotted lines. Relative NH_4_^+^ affinity values represent the relative *E. coli K*_*m*_ for NH_4_^+^ (*K*_A_) divided by that of *R. palustris* (*K*_AR_). For a ratio of 1, each species had a default *K*_*m*_ for NH_4_^+^ of 0.01 mM. To the left of 1, the *R. palustris K*_*m*_ value was raised. To the right of 1, the *E. coli K*_*m*_ value was raised. Both panels show final cell densities (solid lines) of *R. palustris* (*Rp*; purple) and *E. coli* (*Ec*; orange) after simulated 2,000-h batch cultures. Starting cell densities (dashed lines) were based on a 1% dilution of cocultures containing 10% *E. coli.* Download FIG S2, TIF file, 0.2 MB. Copyright © 2017 McCully et al. 2017 McCully et al. This content is distributed under the terms of the Creative Commons Attribution 4.0 International license.

10.1128/mBio.01620-17.10 TABLE S2  Strains, plasmids, and primers used in this study. Download TABLE S2, DOCX file, 0.02 MB. Copyright © 2017 McCully et al. 2017 McCully et al. This content is distributed under the terms of the Creative Commons Attribution 4.0 International license.

The SyFFoN prediction that mutualism stability requires that *E. coli* have a higher affinity for NH_4_^+^ than does *R. palustris* might seem at odds with other models of resource competition, wherein an increased cost of cooperation and/or decreased resource capture by the cooperator (as should be the case when *E. coli* further outcompetes *R. palustris* for NH_4_^+^) can result in extinction of the cooperator (35, 36). We reasoned that the population-level outcome from altering the affinity for a communally valuable nutrient depends on whether the nutrient is generated intra- or extracellularly. Intracellular generation of a communally valuable nutrient would enforce partial privatization, as the producer would have a steep advantage in retaining a sufficient portion of the nutrient pool. No matter what the recipient affinity for the nutrient, it could never overcome the advantage imparted by the physical boundary of the producer’s cell envelope. Extracellular generation, on the other hand, such as the enzymatic release of sugar monomers from extracellular polysaccharides, can result in the majority of the nutrient being lost to neighboring cells, making the ability of the producer to capture the nutrient more important (35, 37). The producer advantage of intracellular nutrient generation is built into SyFFoN, as N_2_ and NH_4_^+^ are treated as two separate nitrogen sources; while both species can acquire extracellular NH_4_^+^, there is also a direct route for N_2_ into an *R. palustris* biomass, bypassing NH_4_^+^ (Fig. 2A; Text S1). To assess whether the intrinsic partial privatization provided by this direct route was responsible for the SyFFoN prediction, we modified SyFFoN so that all N_2_ went through NH_4_^+^ before it could be assimilated by either species (Fig. 2C), mimicking extracellular generation of NH_4_^+^. In this configuration, a disproportionately high affinity for NH_4_^+^ by either species prevented the growth of either one or both species (Fig. 2D). In the range where net growth of both species was predicted, coculture growth was dependent on preferential access by *R. palustris*, the producer rather than the recipient (Fig. 2D), similar to predictions from studies between cooperator and competitor cells (35, 37). Thus, the requirement that the *E. coli* recipient be more competitive for NH_4_^+^ to maintain coexistence is expected to only be true for intracellularly generated NH_4_^+^.

### Genetic disruption of AmtB NH_4_^+^ transporters affects relative affinities for NH_4_^+^.

Bacterial cells generally acquire NH_4_^+^ through two mechanisms: passive diffusion of NH_3_ or uptake by AmtB transporters (Fig. 1B) (24). We hypothesized that deletion of the *amtB* gene in either species would result in a lower affinity for NH_4_^+^ in that species and thus could be used to test how the relative NH_4_^+^ affinity impacts coculture dynamics. We generated ΔAmtB mutants of both *E. coli* and *R. palustris* and first characterized the effects of the mutations in monocultures. Deletion of *amtB* in *E. coli* had no effect on growth or fermentation profiles when 15 mM NH_4_Cl was provided (Fig. S3), consistent with previous observations where ΔAmtB growth defects were only apparent at NH_4_^+^ concentrations below 20 μM (24). In *R. palustris* ΔAmtB monocultures with N_2_ as the nitrogen source, growth trends were equivalent to those of the parent strain; however, *R. palustris* ΔAmtB excreted more NH_4_^+^ than the parent strain and about a third of that excreted by *R. palustris* Nx (Fig. S3C and D). In line with our hypothesis, NH_4_^+^ excretion by *R. palustris* ΔAmtB could be due to a decreased ability to reacquire NH_4_^+^ lost by diffusion, resulting in increased net NH_4_^+^ excretion. Alternatively, we considered that NH_4_^+^ excretion by *R. palustris* ΔAmtB could be due to improper nitrogenase regulation in response to NH_4_^+^ (27, 38). However, we found that nitrogenase activity in *R. palustris* ΔAmtB responded similarly to NH_4_^+^-induced inhibition as in the parental strain (Fig. S4). These observations demonstrated that *R. palustris* ΔAmtB NH_4_^+^ excretion is likely due to a poor ability to reacquire NH_4_^+^ lost by diffusion.

10.1128/mBio.01620-17.4 FIG S3  *E. coli* ΔAmtB and *R. palustris* ΔAmtB monoculture growth and metabolic trends. (A and B) Growth rates (A) and fermentation product yields (B) from WT *E. coli* (filled) or *E. coli* ΔAmtB (open) monocultures grown in MDC with 25 mM glucose and 15 mM NH_4_Cl. Lower concentrations of NH_4_Cl were not tested because other studies have shown that growth is only affected below 20 μM (24). In batch culture, 20 μM would provide insufficient nitrogen to observe any growth. Fermentation profiles were determined from samples from stationary monocultures. Error bars indicate standard deviations (SD; *n* = 3). (C and D) Growth curves (C) and relative NH_4_^+^ excretion (D) of *R. palustris* monocultures grown in MDC with 3 mM sodium acetate and a 100% N_2_ headspace. Error bars indicate SD (*n* = 4). Download FIG S3, TIF file, 0.3 MB. Copyright © 2017 McCully et al. 2017 McCully et al. This content is distributed under the terms of the Creative Commons Attribution 4.0 International license.

10.1128/mBio.01620-17.5 FIG S4  *R. palustris* ΔAmtB responds to NH_4_^+^-induced switch off of nitrogenase. The effect of either NH_4_Cl or NaCl on growth (A and C) and H_2_ production (B, D, and E) by *R. palustris* Nx, ΔAmtB, or parental monocultures. (A to D) *R. palustris* monocultures were grown in MDC with 20 mM sodium acetate and 100% N_2_ headspace until mid-exponential phase, and then cultures were supplemented with either 15 mM NH_4_Cl or 15 mM NaCl at the time indicated by the arrow. Nitrogenase activity was measured using H_2_ production as a convenient alternative to an acetylene reduction assay, since H_2_ is an obligate product of the nitrogenase reaction and, unlike most N_2_-fixing bacteria that oxidize any H_2_ produced, our *R. palustris* strain accumulates H_2_ due to a Δ*hupS* mutation that inactivates hydrogenase. (E) *R. palustris* monocultures were grown in MDC with 20 mM sodium acetate and 100% N_2_ headspace with (gray) or without (white) 15 mM NH_4_Cl. Samples for determining H_2_ yields were taken 1 week after inoculation, within 24 h of entry into stationary phase. Error bars indicate standard deviations (*n* = 3). Download FIG S4, TIF file, 0.3 MB. Copyright © 2017 McCully et al. 2017 McCully et al. This content is distributed under the terms of the Creative Commons Attribution 4.0 International license.

To test our hypothesis that deletion of *amtB* would lower cellular affinity for NH_4_^+^, we directly tested all possible *E. coli* and *R. palustris* strain combinations in competition assays in which ample carbon was available for each species but the NH_4_^+^ concentration was kept low. Specifically, a small amount of NH_4_^+^ was added every hour to bring the final NH_4_^+^ concentration to approximately 5 μM, although it is possible that the NH_4_^+^ concentration exceeded 5 μM at early time points when consumption rates could have been slow due to low cell densities (Fig. 3). In this competition assay, the species that is more competitive for NH_4_^+^ should reach a higher cell density than the other species. In all cases, wild-type (WT) *E. coli* was more competitive for NH_4_^+^ than any *R. palustris* strain. However, each *R. palustris* strain was able to outcompete *E. coli* ΔAmtB (Fig. 3), even though the *E. coli* maximum growth rate is 4.6 times higher than that of *R. palustris* (Fig. S3). Even *R. palustris* strains lacking AmtB outcompeted *E. coli* ΔAmtB (Fig. 3), indicating that *R. palustris* has a higher affinity for NH_4_^+^ than *E. coli*, independent of AmtB. These data confirmed that deletion of *amtB* was an effective means by which to lower the relative affinity for NH_4_^+^ in each mutualistic partner.

### Alteration of relative NH_4_^+^ affinities affects mutualistic partner frequencies.

**FIG 4:**
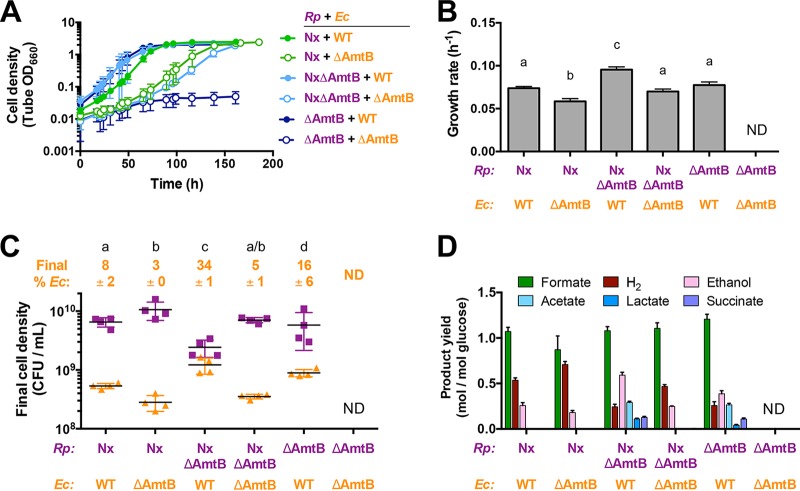
AmtB influences population and metabolic trends of both partners in coculture. Growth curves (A), growth rates (B), final cell densities (C), and fermentation product yields (D) from cocultures of all combinations of mutants lacking AmtB are shown. Final cell densities and fermentation product yields were determined after 1 week, within 24 h of entering stationary phase. Cocultures were started with a 1% inoculum of stationary starter cocultures grown from single colonies that reached comparable final cell densities, as those shown in panels A and C. ND, not determined. Error bars indicate standard deviations (*n* = 4). Different letters indicate statistical differences (*P* < 0.05, determined by a one-way analysis of variance with Tukey’s multiple-comparisons posttest).

We then examined how relative affinities for excreted NH_4_^+^ influenced mutualism dynamics by comparing the growth trends of cocultures containing either WT *E. coli* or *E. coli* ΔAmtB, paired with either *R. palustris* ΔAmtB, *R. palustris* Nx, or *R. palustris* NxΔAmtB, the latter of which we previously determined exhibited 3-fold-higher NH_4_^+^ excretion levels than the Nx strain in monoculture (28). We did not use the *R. palustris* parent strain, because it was previously determined not to support coculture growth due to insufficient NH_4_^+^ excretion (28). For each *R. palustris* partner, cocultures with *E. coli* ΔAmtB grew slower than cocultures with WT *E. coli* (Fig. 4A and B). *E. coli* ΔAmtB also constituted a lower percentage of the population and achieved lower cell densities than did WT *E. coli* when paired with the same *R. palustris* strain (Fig. 4C). These lower frequencies were consistent with the competitive disadvantage of *E. coli* ΔAmtB for excreted NH_4_^+^ (Fig. 3). AmtB is only expected to be important for NH_4_^+^ acquisition when concentrations are below 20 μM (24). In agreement with this expectation, supplementing cocultures with 15 mM NH_4_Cl led to rapid growth and domination by *E. coli* ΔAmtB (Fig. S5), which resembled those characteristics of previous cocultures with WT *E. coli* that were supplemented with 15 mM NH_4_Cl (28). The low final cell density in cocultures with 15 mM NH_4_Cl (Fig. S5) is due to rapid organic acid excretion associated with the high *E. coli* growth rate, which leads to culture acidification that prevents *R. palustris* growth (28).

10.1128/mBio.01620-17.6 FIG S5  *E. coli* ΔAmtB outgrows *R. palustris* Nx and prevents *R. palustris* growth when cocultures are supplemented with 15 mM NH_4_Cl. Growth curves (A), final cell densities (B), and product yields (C) from cocultures of *E. coli* ΔAmtB paired with *R. palustris* Nx supplied with either 15 mM NaCl (gray) or NH_4_Cl (black). Final cell densities were taken within 24 h into stationary phase. Initial cell densities were approximately 3 × 10^6^ CFU/ml for *E. coli* ΔAmtB and 1 × 10^8^ CFU/ml for *R. palustris* Nx, which represented a 1% inoculum from stationary starter cocultures from single colonies. Error bars indicate standard deviations (*n* = 3). Download FIG S5, TIF file, 0.2 MB. Copyright © 2017 McCully et al. 2017 McCully et al. This content is distributed under the terms of the Creative Commons Attribution 4.0 International license.

For *R. palustris* strains lacking AmtB, the effects on population trends varied. Consistent with our previous work, *R. palustris* NxΔAmtB supported higher WT *E. coli* percentages and cell densities (Fig. 4C) (28). Similar to adding 15 mM NH_4_^+^, the high NH_4_^+^ excretion level from *R. palustris* NxΔAmtB (Fig. S3D) resulted in faster *E. coli* growth and accumulation of consumable organic acids (acetate, succinate, and lactate), which acidify the medium and inhibit *R. palustris* growth (Fig. 4D) (28). Surprisingly, although *R. palustris* ΔAmtB excreted the least amount of NH_4_^+^ in monoculture, it supported a higher WT *E. coli* population in coculture, and consumable organic acids accumulated (Fig. 4C and D). These trends resembled those from cocultures with *R. palustris* NxΔAmtB (Fig. 4C and D). Unlike Nx strains, which have constitutive nitrogenase activity due to a mutation in the transcriptional activator *nifA* (32), *R. palustris* ΔAmtB has WT *nifA*. Thus, *R. palustris* ΔAmtB can likely still regulate nitrogenase expression, and thereby its activity, in response to nitrogen starvation. We hypothesized that in coculture with WT *E. coli*, *R. palustris* ΔAmtB might experience heightened nitrogen starvation, as NH_4_^+^ consumption by WT *E. coli* would limit NH_4_^+^ reacquisition by *R. palustris* ΔAmtB (in an *R. palustris* ΔAmtB monoculture, any lost NH_4_^+^ would simply benefit its clones). We therefore tested whether coculture conditions stimulated higher nitrogenase activity by using an acetylene reduction assay. In agreement with our hypothesis, *R. palustris* ΔAmtB had increased nitrogenase activity under coculture conditions compared to monocultures, whereas *R. palustris* Nx, which exhibits constitutive nitrogenase activity, showed similar levels under both conditions (Fig. S6). Thus, the relatively greater WT *E. coli* population in coculture with *R. palustris* ΔAmtB was likely due to both the competitive advantage for acquiring NH_4_^+^ over *R. palustris* ΔAmtB (Fig. 3) and the higher NH_4_^+^ cross-feeding levels associated with increased nitrogenase activity.

10.1128/mBio.01620-17.7 FIG S6  *R. palustris* ΔAmtB nitrogenase activity increases in coculture. Normalized nitrogenase activity of *R. palustris* in monoculture (Mono) or coculture (Co) was measured in an acetylene reduction assay. Ethylene levels were divided by total *R. palustris* CFU in the test tube and then normalized to the *R. palustris* Nx monoculture value. Error bars indicate standard deviations (*n* = 4). *, statistical difference between monoculture and coculture conditions (*P* < 0.05, determined using multiple two-tailed *t* tests); ns, no significant difference. Download FIG S6, TIF file, 0.1 MB. Copyright © 2017 McCully et al. 2017 McCully et al. This content is distributed under the terms of the Creative Commons Attribution 4.0 International license.

### *E. coli* must have a competitive advantage for NH_4_^+^ acquisition to avoid mutualism collapse.

Unlike all other pairings, cocultures of *R. palustris* ΔAmtB paired with *E. coli* ΔAmtB showed little growth when started from a single colony of each species (Fig. 4A), a method that we routinely use to initiate cocultures (28, 33). We reasoned that the higher *R. palustris* ΔAmtB affinity for NH_4_^+^ relative to *E. coli* ΔAmtB (Fig. 3) likely led to community collapse, as predicted by SyFFoN (Fig. 2B). Even though SyFFoN predicted *R. palustris* growth when outcompeting *E. coli* for NH_4_^+^ (Fig. 2B), SyFFoN likely underestimated the time required to achieve these densities, if they would be achieved at all, as SyFFoN does not take into account cell death, which is known to occur when *E. coli* growth is prevented (33). Consistent with the hypothesis that poor coculture growth was due to a competitive disadvantage of *E. coli* ΔAmtB for NH_4_^+^, SyFFoN simulations indicated that starting with a more dilute *R. palustris* inoculum would increase the probability that any given *E. coli* ΔAmtB cell would acquire NH_4_^+^ when in competition with *R. palustris* and thereby overcome the competitive disadvantage of *E. coli* ΔAmtB for NH_4_^+^ (Fig. S7). Indeed, we observed greater growth of both species when cocultures were inoculated at ratios with equal or higher relative densities of *E. coli* ΔAmtB versus *R. palustris* ΔAmtB (Fig. S7).

10.1128/mBio.01620-17.8 FIG S7  Higher initial cell densities of *E. coli* ΔAmtB can partially compensate for a low *E. coli* NH_4_^+^ affinity. Simulations (A) and empirical data (B and C) showing the effects of the initial CFU ratio of *R. palustris* (*Rp*) to *E. coli* (*Ec*) on population and coculture growth trends are shown for *E. coli*, with a lower affinity for NH_4_^+^ than *R. palustris*. (A) Batch cultures (300 h) were simulated with a relative *R. palustris*:*E. coli* affinity ratio (*Rp*:*Ec*) for NH_4_^+^ of 1000 (K_A_ = 10; K_AR_ = 0.01). (B and C) Changes in cell densities after 1 week of growth (B) and growth curves of cocultures inoculated at different species ratios (C). In all three panels, a ratio of 1 represents 2.7 × 10^6^ cells/ml (A) or CFU/ml (B and C). Thus, the listed ratios were achieved by diluting either species rather than concentrating them. Error bars indicate standard deviations (*n* = 3). Download FIG S7, TIF file, 0.2 MB. Copyright © 2017 McCully et al. 2017 McCully et al. This content is distributed under the terms of the Creative Commons Attribution 4.0 International license.

**FIG 5:**
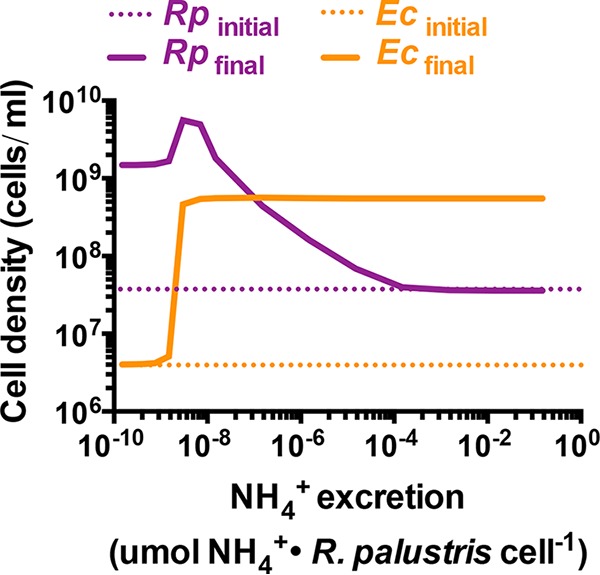
Higher *R. palustris* NH_4_^+^ excretion levels are predicted to compensate for a low *E. coli* NH_4_^+^ affinity. Batch cultures (after 300 h) were simulated with a relative NH_4_^+^ affinity of 1,000 (*R. palustris*:*E. coli* affinity ratio [*Rp*:*Ec*]; affinity values are the inverse of *K_m_* values) over different *R. palustris* NH_4_^+^ excretion levels (SyFFoN parameter R_A_). Final cell densities, solid lines; initial cell densities, dotted lines.

The explanation that mutualism collapse was due to a competitive advantage of *R. palustris* ΔAmtB over *E. coli* ΔAmtB for NH_4_^+^ called into question why cocultures pairing *E. coli* ΔAmtB with either *R. palustris* Nx or *R. palustris* NxΔAmtB did not collapse as well (Fig. 4), given that in all of these pairings *E. coli* ΔAmtB was at a competitive disadvantage (Fig. 3). We hypothesized that a relatively high NH_4_^+^ excretion level by these latter *R. palustris* strains (Fig. S3D) could compensate for a low *E. coli* NH_4_^+^ affinity. To explore this hypothesis, we simulated cocultures with the *R. palustris* affinity for NH_4_^+^ set high relative to that of *E. coli* (*R. palustris*:*E. coli* affinity ratio, 1,000) and varied the *R. palustris* NH_4_^+^ excretion level (Fig. 5). Indeed, increasing *R. palustris* NH_4_^+^ excretion was predicted to overcome a low *E. coli* affinity for NH_4_^+^ and support growth of both species (Fig. 5). The only exception was at the highest levels of NH_4_^+^ excretion, where *R. palustris* growth was predicted to be inhibited due to rapid *E. coli* growth and subsequent accumulation of organic acids that acidify the environment (Fig. 5), similar to previous observations where we experimentally increased the NH_4_^+^ excretion level (28). These simulations suggested that *R. palustris* Nx and NxΔAmtB supported coculture growth with *E. coli* ΔAmtB due to higher NH_4_^+^ excretion levels (Fig. S3D), whereas a combination of low NH_4_^+^ excretion by *R. palustris* ΔAmtB (Fig. S3D) and a low affinity for NH_4_^+^ by *E. coli* ΔAmtB led to collapse of the mutualism in this pairing.

**FIG 6:**
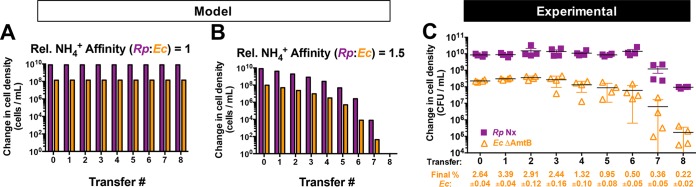
A low *E. coli* affinity for NH_4_^+^ results in coculture collapse over serial transfers when paired with *R. palustris* Nx. (A and B) Simulated batch cultures (300 h) were serially transferred using a 1% inoculum based on the cell density at 300 h for the previous culture. Relative NH_4_^+^ affinity values represent the relative *E. coli K*_*m*_ for NH_4_^+^ (*K*_A_) divided by that of *R. palustris* (*K*_A_). *K*_A_ and *K*_AR_ were both 0.01 mM in panel A. *K*_A_ was 0.015 mM and *K*_AR_ was 0.01 mM in panel B. (C) Change in cell densities of *R. palustris* Nx and *E. coli* ΔAmtB of cocultures grown for 1 week, less than 24 h into stationary phase. A 1% inoculum was used for each subsequent serial transfer. Error bars indicate standard deviations (SD; *n* = 4). Final *E. coli* cell percentages ± SD for each transfer are shown.

To this point, we had only considered the effect of severe discrepancies in NH_4_^+^ affinities between the two species (e.g., a 1,000-fold difference in *K*_*m*_ values in our simulations) as a mechanism leading to coculture collapse within the time period of a single culturing. However, we wondered if a subtle discrepancy in NH_4_^+^ affinities could lead to coculture collapse if given more time. We therefore simulated serial transfers of cocultures with partners having different relative NH_4_^+^ affinities (Fig. 6A and B). At equivalent NH_4_^+^ affinities (Fig. 6A), both species were predicted to be maintained over serial transfers. However, when the relative affinities approached a threshold (relative *R. palustris*:*E. coli* affinity ratio, 1.5), cell densities of both species were predicted to decrease over serial transfers (Fig. 6B). This decline in coculture growth is due to *E. coli* being slowly but progressively outcompeted for NH_4_^+^ by *R. palustris*. As the difference between the *R. palustris* and *E. coli* populations expands, *R. palustris* cells have a greater chance of acquiring NH_4_^+^ than the smaller *E. coli* population, further starving *E. coli* and simultaneously cutting off *R. palustris* from its supply of organic acids from *E. coli*.

The above prediction prompted us to investigate if cocultures pairing *R. palustris* Nx with *E. coli* ΔAmtB were stable through serial transfers. We focused on cocultures with *R. palustris* Nx rather than *R. palustris* NxΔAmtB, because *R. palustris* Nx has AmtB and would therefore be most likely to outcompete *E. coli* ΔAmtB. Strikingly, over eight serial transfers of cocultures pairing *R. palustris* Nx with *E. coli* ΔAmtB, we observed a significant decrease in cell densities of both partners (Fig. 6C). This decline in coculture growth over serial transfers was in stark contrast to results with cocultures of *R. palustris* Nx paired with WT *E. coli*, which we have serially transferred over 100 times with no extinction events (J. B. McKinlay, unpublished data). These results indicate that the recipient population must have a competitive advantage for a cross-fed nutrient relative to the producer population to avoid mutualism collapse.

## DISCUSSION

Here, we demonstrated that within a mutualistic relationship, partners can compete for a cross-fed nutrient upon which the mutualistic interaction is based, in this case NH_4_^+^. This competition can impact partner frequencies and mutualism stability. We demonstrated that efficient nutrient reacquisition by the producer can render nutrient excretion levels insufficient for mutualistic growth, starving the recipient and leading to tragedy of the commons (Fig. 6) (39). Conversely, recipient-biased competition for a cross-fed nutrient promotes mutualism stability. As noted above, the importance of this recipient-biased competitive advantage likely depends on whether the communally valuable resource is generated intracellularly or extracellularly (compare Fig. 2A and C). Intracellular synthesis ensures that a portion of the nutrient pool can be assimilated by the producing partner regardless of the differential affinity between the partners for that nutrient after excretion (Fig. 2A). Intracellular generation therefore helps stabilize a mutualism against an otherwise-competitive recipient by enforcing partial privatization. The competitive advantage of the recipient is in turn necessary to limit reacquisition of the excreted nutrient by the producer and thereby to drive directionality in nutrient exchange. Although partial privatization has primarily been thought to depend on mechanisms used by the producer to retain a portion of a communally valuable resource (16), our results indicate that the degree of privatization can be influenced by the partner as well; competition for the excreted nutrient pool impacts how much of a cross-fed resource will be shared versus reacquired. In effect, recipient-biased competition for an excreted communally valuable nutrient avoids tragedy of the commons by enforcing partial privatization over complete privatization.

It is expected that for mutualistic relationships based on the extracellular generation of nutrients, such as the release of sugar from a polymer, a high affinity for the nutrient by either partner can collapse the mutualism (Fig. 2D). It has been shown that microbes that excrete sugar polymer-degrading enzymes in the presence of competitors must have an advantage in obtaining the released sugars to proliferate, or even to avoid extinction (35–37). Supplementing a mutualism with an exogenous source of an otherwise-excreted communally valuable nutrient could also be viewed to mimic extracellular production. In these cases, the population outcome is also heavily influenced by the competitive affinities of each partner. For example, progressively adding exogenous nutrients to a yeast coculture stabilized by amino acid cross-feeding was shown to shift a mutualistic relationship to one of competition (21).

The importance of the recipient having the upper hand in interpartner competition likely applies to other synthetic cocultures and natural microbial mutualisms that are based on the cross-feeding of communally valuable nutrients that are generated intracellularly, including amino acids (21, 40, 41) and vitamin B_12_ (7, 12). The same rule could also apply to interkingdom and nonmicrobial cross-feeding examples, such as those between plants and bacteria, fungi, or pollinators (1). In these cases, any decrease in resource release or emergence of traits allowing for reacquisition of a released resource would be expected to undermine the mutualism. Conversely, some nonmicrobial examples of cooperative feeding would be expected to follow the predictions for microbial mutualisms based on the extracellular generation of a communally valuable resource. For example, cooperative hunting between grouper fish and moray eels (42) or cooperative harvesting of honey from bee hives between honeyguide birds and humans (43) would be expected to collapse if a single partner were to monopolize the resource (44). Indeed, the cooperative relationship between honeyguide birds and humans has declined in areas that have adopted bee-keeping practices, though in this case such declines are due to a technological advancement rather than evolution (43).

Our study also provided mechanistic insights into acquisition of communally valuable nutrients. AmtB transporters were shown to be crucial determinants of interpartner competition for NH_4_^+^. We were intrigued to find that when both species lacked AmtB, *R. palustris* outcompeted *E. coli* for NH_4_^+^ (Fig. 5), enough to collapse the mutualism within a single culturing (Fig. 3). Whether by maximizing NH_4_^+^ retention or reacquisition, *R. palustris*, and perhaps other N_2_ fixers, might have additional mechanisms aside from AmtB to minimize loss of NH_4_^+^ as NH_3_. These mechanisms could include a relatively low internal pH to favor NH_4_^+^ over NH_3_, negatively charged surface features, or relatively high affinities by NH_4_^+^-assimilating enzymes, such as glutamine synthetase. There are several reasons why it would be beneficial for N_2_ fixers to minimize NH_4_^+^ loss. First, N_2_ fixation is expensive, both in terms of the enzymes involved (45) and the reaction itself, costing 16 ATP to convert 1 N_2_ into 2 NH_3_ (46). Passive loss of NH_3_ would only add to this cost, as more N_2_ would have to be fixed to compensate. Second, loss of NH_4_^+^ could benefit nearby microbes competing against an N_2_ fixer for separate limiting nutrients (15, 47). The possibility that N_2_ fixers could have a superior ability to retain or acquire NH_4_^+^, perhaps by using mechanisms that are independent of AmtB, is not far-fetched. Bacteria are known to exhibit differential mechanisms to compete for nutrients. For example, iron acquisition commonly involves the excretion of iron-binding molecules or proteins called siderophores, which can differ in chemical structure and affinity for iron. These structural differences also influence their potential to be utilized by competitors and therefore their communal value as an extracellularly generated resource (48). Strategies to utilize siderophores as a shared resource are numerous, and they lead to different cooperative or competitive outcomes in microbial communities (48, 49). One must consider that additional mechanisms for acquiring NH_4_^+^ beyond AmtB might likewise exist. Understanding the physiological mechanisms that confer competitive advantages for nutrient acquisition between species will undoubtedly aid in describing the interplay between competition and cooperation within mutualisms.

## MATERIALS AND METHODS

### Strains and growth conditions.

Strains, plasmids, and primers are listed in Table S2. All *R. palustris* strains contained *ΔuppE* and *ΔhupS* mutations to facilitate accurate CFU measurements by preventing cell aggregation (50) and to prevent H_2_ uptake, respectively. *E. coli* was cultivated on Luria-Burtani (LB) agar, and *R. palustris* was cultivated on defined mineral (PM) agar (51) with 10 mM succinate. (NH_4_)_2_SO_4_ was omitted from PM agar for determining *R. palustris* CFU. Monocultures and cocultures were grown in 10 ml of defined M9-derived coculture medium (MDC) (28) in 27-ml anaerobic test tubes. To make the medium anaerobic, MDC was exposed to N_2_ via bubbling, and then tubes were sealed with rubber stoppers and aluminum crimps and then autoclaved. After autoclaving, MDC medium was supplemented with cation solution (1% [vol/vol]; 100 mM MgSO_4_ and 10 mM CaCl_2_ stock concentration) and glucose (25 mM final concentration), unless indicated otherwise. *E. coli* monocultures were also supplemented with 15 mM NH_4_Cl. All cultures were grown at 30°C laying horizontally under a 60-W incandescent bulb with shaking at 150 rpm. Starter cocultures were inoculated with 200 μl MDC containing a suspension of a single colony of each species. Test cocultures were inoculated using a 1% inoculum from starter cocultures. Serial transfers were also inoculated with a 1% inoculum. Kanamycin and gentamicin were added to final concentrations of 100 μg/ml for cultures of *R. palustris* and 15 μg/ml for *E. coli* cultures when appropriate.

### Generation of *R. palustris* mutants.

*R. palustris* mutants were derived from wild-type CGA009 (52). Generation of strains CGA4004, CGA4005, and CGA4021 is described elsewhere (28). To generate strain CGA4026 (*R. palustris* ΔAmtB), the WT *nifA* gene was amplified using primers JBM1 and JBM2, digested with XbaI and BamHI, and ligated into plasmid pJQ200SK to make pJQnifA16. This suicide vector was then introduced into CGA4021 by conjugation, and sequential selection and screening were performed as described (53) to replace nifA* with WT *nifA*. Reintroduction of the WT *nifA* gene was confirmed by PCR and sequencing.

### Generation of the *E. coli* ΔAmtB mutant.

P1 transduction (54) was used to introduce ΔamtB::*km* from the Keio Collection strain JW0441-1 (55) into *E. coli* MG1655. The ΔamtB::*km* genotype of kanamycin-resistant colonies was confirmed by PCR and sequencing.

### Analytic procedures.

Cell density was assayed based on the optical density at 660 nm (OD_660_) using a Genesys 20 visible spectrophotometer (Thermo-Fisher, Waltham, MA). Growth curve readings were obtained in culture tubes without sampling (i.e., tube OD_660_). Specific growth rates were determined using OD_660_ readings between 0.1 and 1.0, a range for which there is a linear correlation between cell density and OD_660_. Final OD_660_ measurements were taken in cuvettes, and samples were diluted into the linear range as necessary. H_2_ was quantified using a gas chromatograph (Shimazu, Kyoto, Japan) with a thermal conductivity detector as described (56). Glucose, organic acids, formate, and ethanol were quantified using a Shimadzu high-performance liquid chromatograph as described (57). NH_4_^+^ was quantified using an indophenol colorimetric assay as described (28). Acetylene reduction assays (45) were performed by first harvesting cells from 10 ml of medium and resuspending in 10 ml of fresh MDC medium in 27-ml sealed tubes preflushed with argon gas. Suspensions were incubated in light for 1 h at 30°C to recover. Then, 250 μl of 100% acetylene gas was injected into the headspace to initiate the assay, and ethylene production was measured over time by gas chromatography, as described (45). Ethylene levels were normalized to total *R. palustris* CFU in the 10-ml volume.

### NH_4_^+^ competition assay.

Fed batch cultures were prepared in custom anaerobic 75-ml serum vials with side sampling ports. Each vial contained a stir bar and 30 ml of MDC and was sealed at both ends with rubber stoppers and aluminum crimps. Each vial was supplemented with 25 mM glucose, 1% (vol/vol) cation solution, and 20 mM sodium acetate. Unlike acetic acid, which *E. coli* excretes, sodium acetate does not change the pH of the medium. Starter monocultures of each species were grown to equivalent CFU (per milliliter) in MDC containing limiting nutrients (3 mM sodium acetate for *R. palustris* and 1.5 mM NH_4_Cl for *E. coli*), and 1 ml of each species culture was inoculated into the serum vials. These competition cocultures were incubated at 30°C under a 60-W incandescent bulb with stirring at 200 rpm for 96 h. Each serum vial was constantly flushed with Ar to maintain anaerobic conditions. NH_4_Cl was fed from a 500 μM NH_4_Cl stock via a peristaltic pump on an automatic timer at a rate of 0.33 ml/min once an hour for a final concentration of ~5 μM upon each addition. The NH_4_^+^ concentration was below the known concentration at which AmtB transporters become important for NH_4_^+^ uptake (24). Samples were taken at 0 and 96 h for quantification of CFU.

### Mathematical modeling.

A Monod model describing bidirectional cross-feeding in batch cultures, called SyFFoN v3 (syntrophy between fermenter and fixer of nitrogen, version 3), was modified from our previous model (33) to allow for competition between *E. coli* and *R. palustris* for NH_4_^+^ as follows: (i) an equation for the *R. palustris* growth rate on NH_4_^+^ was added to boost the *R. palustris* growth rate when acquiring NH_4_^+^ and (ii) the ability for *R. palustris* to consume NH_4_^+^ was added along with an *R. palustris K*_*m*_ for NH_4_^+^ (*K*_AR_). Default NH_4_^+^ *K*_*m*_ values were set to 0.01 mM for both species, to achieve a ratio of 1. To achieve higher *R. palustris* or *E. coli* relative NH_4_^+^ affinities, the *E. coli* or *R. palustris K*_*m*_ value was raised, respectively. Simulated cultures were run for 300 h unless noted otherwise. Normally, full glucose consumption occurs by ~100 h under typical experimental conditions and in corresponding simulations, but 300 h was allowed to capture trends that would take longer to emerge in response to parameter changes while still approximating a reasonable experimental time frame. Equations and default parameter values derived from our experimental data can be found in Text S1 and Table S1. SyFFoN v3 is run in RStudio and is available for download at https://github.com/McKinlab/Coculture-Mutualism.
