# Supplementary material for: Recipient-Biased Competition for an Intracellularly Generated Cross-Fed Nutrient Is Required for Coexistence of Microbial Mutualists
Source: mBio. 2017 Nov 28;8(6):e01620-17. doi: 10.1128/mBio.01620-17 (PMC5705916; doi:10.1128/mBio.01620-17)
Supplement: TABLE S1 [file mbo006173615st1.docx]

| **Parameter** | **Value** | **Description (Units); Source** |
| --- | --- | --- |
| μ_EcMAX_ | 0.2800 | *E. coli* max growth rate (h^-1^); Monoculture |
| μ_RpMAX_ | 0.0772 or *0*^a^ | *R. palustris* max growth rate (h^-1^); Monoculture |
| μ_RpMAX2_ | 0.0152 or  *0.0772* ^b^ | Boost on *R. palustris* growth rate in presence of NH_4_^+^ (h^-1^); Monoculture^b^ |
| G | 25 | Glucose (mM) |
| A | 0.00005 | NH_4_^+^ (mM); from initial (NH_4_)_6_Mo_7_O_24_·4H_2_O concentration |
| C | 0 | Consumable organic acids (those that *R. palustris* was observed to consume: lactate, acetate, and succinate; mM) |
| N | 70 | N_2_ (assumed to be fully dissolved; mM) |
| f | 0 | Formate (mM) |
| e | 0 | Ethanol (mM) |
| CO2 | 0 | Carbon dioxide (mM) |
| K_G_ | 0.02 | *E. coli* affinity (Michaelis-Menten constant (K_m_)) for glucose (mM); (1) |
| K_C_ | 0.01 | *R. palustris* affinity (K_m_) for consumable organic acids (mM); Assumed |
| K_A_ | 0.01 | *E. coli* affinity for NH_4_^+^ (mM); (2) |
| K_AR_ | 0.01 | *R. palustris* affinity for NH_4_^+^ (mM); Assumed^c^ |
| K_N_ | 6 | *R. palustris* affinity (K_m_) for N_2_ (mM) |
| Ec | 0.4 x 10^7^ | *E. coli* cell density (cells / ml) |
| Rp | 3.6 x 10^7^ | *R. palustris* cell density (cells / ml) |
| b_Ec_ | 10^43^ | Resistance of *E. coli* to low pH (mM) |
| b_Rp_ | 10^32^ | Resistance of *R. palustris* to low pH (mM) |
| Y_G_ | 8 x 10^7^ | Glucose-limited *E. coli* growth yield (cells / μmol glucose); Glucose-limited *E. coli* culture |
| Y_A_ | 1 x 10^9^ | NH_4_^+^-limited *E. coli* growth yield (cells / μmol NH_4_^+­^); NH_4_^+^-limited *E. coli* culture |
| Y_C_ | 2.5 x 10^8^ | Organic acid-limited *R. palustris* growth yield (cells / μmol organic acid); Acetate-limited *R. palustris* culture |
| Y_N_ | 5 x 10^8^ | N_2_-limited *R. palustris* growth yield cells / μmol N_2_; N_2_-limited *R. palustris* culture |
| R_C_ | 1.9 x 10^-8^ | Fraction of glucose converted to organic acids (μmol glucose / cell) |
| R_f_ | 8 x 10^-9^ | Fraction of glucose converted to formate (μmol glucose / cell) |
| R_e_ | 4.5 x 10^-9^ | Fraction of glucose converted to ethanol (μmol glucose / cell) |
| R_CO2_ | 5 x 10^-10^ | Fraction of glucose converted to CO_2_ (μmol glucose / cell) |
| R_HRp_ | 2 x 10^-9^ | *R. palustris* H_2_ production (μmol H_2_ / *R. palustris* cell) |
| R_HEc_ | 5 x 10^-9^ | *E. coli* H_2_ production (μmol H_2_ / *E. coli* cell) |
| R_A_ | 0.15 x 10^-9^ or  1.5 x 10^-9 d^ | *R. palustris* NH_4_^+^ production (μmol NH_4_^+^ / cell) ^d^ |
| r_C_ | 300 x 10^-11^ | *E. coli* specific growth-independent rate of glucose conversion to consumable organic acids (μmol glucose / cell / h) (3) |
| r_f_ | 47 x 10^-11^ | *E. coli* specific growth-independent rate of glucose conversion to formate (μmol glucose / cell / h) (3) |
| r_e_ | 15 x 10^-11^ | *E. coli* specific growth-independent rate of glucose conversion to ethanol (μmol glucose / cell / h) (3) |
| r_CO2_ | 2 x 10^-11^ | *E. coli* specific growth-independent rate of glucose conversion to CO_2_ (μmol glucose / cell / h) (3) |
| r_H_ | 2 x 10^-11^ | *E. coli* specific growth-independent rate of H_2_ production (μmol H_2_ / cell / h) (3) |
| r_C_mono_ | 1.2 x 10^-11^ | *E. coli* specific growth-independent rate of glucose conversion to consumable organic acids when consumable organic acids accumulate (μmol glucose / cell / h); (4) |
| r_f_mono_ | 0.83 x 10^-11^ | *E. coli* specific growth-independent rate of glucose conversion to formate when consumable organic acids accumulate (μmol glucose / cell / h); (4) |
| r_e_mono_ | 0.5 x 10^-11^ | *E. coli* specific growth-independent rate of glucose conversion to ethanol when consumable organic acids accumulate (μmol glucose / cell / h); (4) |
| r_co2_mono_ | 1.3 x 10^-11^ | *E. coli* specific growth-independent rate of glucose conversion to CO_2_ when consumable organic acids accumulate (μmol glucose / cell / h); (4) |
| r_H_mono_ | 0.83 x 10^-11^ | *E. coli* specific growth-independent rate of glucose conversion to H_2_ when consumable organic acids accumulate (μmol glucose / cell / h); (4) |
| r_Hp_ | 27 x 10^-11^ | *R. palustris* specific growth-independent rate of H_2_ production (μmol H_2_ / cell / h) |

^a^ In the alternative model shown in Figure 2C, this value is set to 0 h^-1^ to prevent direct utilization of N_2_.

^b^ Default increased growth rate by 0.0152 h^-1^ in presence of NH_4_^+^ versus N_2_ based on the difference in experimentally determined growth rates in *R. palustris* monocultures grown with either NH_4_^+^ or N_2_ as a nitrogen source. In the alternative model shown in Figure 2C when μ_RpMAX_ is set to 0 h^-1^, this value was changed to 0.0772 h^-1^ to reflect the growth rate during N_2_ fixation

^c^ K_AR_ was assumed to be equivalent to the published *E. coli* K_m_ (2) for NH_4_^+^ (K_A_).

^d^ R_A_ is the net NH_4_^+^ excreted per *R. palustris* cell derived from empirical measurements; the actual conversion of N_2_ to NH_4_^+^ is expected to be much higher as only a portion is excreted and the rest is incorporated into *R. palustris* biomass. In the alternative model, NH_4_^+^ excretion (R_A_) was increased to 1.5*10^-9^ to account for all N_2_ being converted to NH_4_^+^ without a direct route from N_2_ to *R. palustris* biomass in this model and to simulate batch culture times resembling those of the default model (i.e., complete glucose consumption within ~ 100 h).

Table S1 references.

1. Buhr A, Daniels GA, Erni B. 1992. The glucose transporter of *Escherichia coli*: Mutants with impaired translocation activity that retain phosphorylation activity. J. Biol. Chem. 267:3847–3851.

2. Khademi S, O’Connell 3rd J, Remis J, Robles-Colmenares Y, Miercke LJ, Stroud RM. 2004. Mechanism of ammonia transport by Amt/MEP/Rh: structure of AmtB at 1.35 A. Science. 305:1587–1594.

3. McCully AL, LaSarre B, Mckinlay JB. 2017. Growth-independent cross-feeding modifies boundaries for coexistence in a bacterial mutualism. Environ. Microbiol. doi:10.1111/1462-2920.13847

4. LaSarre B, McCully AL, Lennon JT, McKinlay JB. 2017. Microbial mutualism dynamics governed by dose-dependent toxicity of cross-fed nutrients. ISME J 11:337–348.
