# Supplementary material for: Recipient-Biased Competition for an Intracellularly Generated Cross-Fed Nutrient Is Required for Coexistence of Microbial Mutualists
Source: mBio. 2017 Nov 28;8(6):e01620-17. doi: 10.1128/mBio.01620-17 (PMC5705916; doi:10.1128/mBio.01620-17)
Supplement: TEXT S1 [file mbo006173615s1.docx]

**SyFFoN_v3 description.**

Equations 1 – 4 were used to describe *E. coli* and *R. palustris* growth rates:

Eq. 1: *E. coli* growth rate; **μ_Ec_** = μ_EcMAX_•[G/(K_G_+G)]•[A/(K_A_+A)]•[b_Ec_/(b_Ec_+10^(f+C)^)]

Eq. 2: *R. palustris* growth rate (N_2_); **μ_Rpn_** = μ_RpMAX_•[C/(K_C_+C)]•[N/(K_N_+N)]•[b_Rp_/(b_Rp_+10^(f+C)^)]

*In the alternative model,* μ_RpMAX_ *was set to zero, eliminating any influence of Eq. 2, which calculates R. palustris growth rate as a function of N_2_ availability,* *on the simulations.*

Eq. 3: *R. palustris* growth rate (NH_4_^+^); **μ_Rpa_** =

μ_RpMAX2_•[C/(K_C_+C)]•[A/(K_AR_+A)]•[b_Rp_/(b_Rp_+10^(f+C)^)]

Eq 4: Total *R. palustris* growth rate; **μ_Rp_** = μ_Rpn_+ μ_Rpa_

Equations 5-14 were used to describe temporal changes in cell densities and extracellular compounds. Numerical constants in product excretion equations are used to account for molar stoichiometric conversions. Numerical constants used in sigmoidal functions are based on those values that resulted in simulations resembling empirical trends. All R and r parameters are expressed in terms of glucose consumed except for R_A,_ which is the amount of NH_4_^+^ produced per *R. palustris* cell (Table S1).

Eq. 5: Glucose; ***d*G/*d*t** = -μ_Ec_•Ec/Y_G_ - μ_Ec_•Ec•(R_c_+R_f_+R_e_+R_CO2_) -

Ec•(G/(K_G_+G))•(10/(10+1.09^(1000• μEc)^))•(b_Ec_/(b_Ec_+10^(f+C)^))•((100/(100+6^C^))• (r_C_+r_f_+r_e_+r_CO2_) + r_C_mono_ + r_f_mono_ + r_e_mono_ + r_CO2_mono_)

Eq. 6: N_2_; ***d*N/*d*t** = -μ_Rp_•Rp•0.5•Ra•(1-(40/(40+1.29^N^)) **- μ_Rp_•Rp/Y_N_**

*In the alternative model (Fig 2C), the term* ***(- μ_Rp_•Rp/Y_N_****) in Eq. 6 was not used thereby preventing N_2_ from going directly into R. palustris biomass.*

Eq. 7: Consumable organic acids; ***d*C/*d*t** = Ec•μ_Ec_•R_c_•2 + Ec•2•(G/( K_G_ +G))

•(10/(10+1.09^(1000• μEc)^))•(b_Ec_ /( b_Ec_ +10^(f+C)^))•(r_C_•(100/(100+6^C^))+r_C_mono_) - (μ_Rp_ •Rp/Y_C_)

– 0.25•Rp•μ_Rp_•Rh_Rp_ - 0.25•Rp•r_Hp_•(C/(K_C_+C))•(40/(40+1.29^N^))•(b_Rp_/( b_Rp_ +10^(f+C)^))

Eq. 8: Formate; ***d*f/*d*t** = (Ec•μ_Ec_•R_f_•6) + Ec•6• (G/(K_G_+G)) • (10/(10+1.09^(1000•^ ^μEc)^))

• (b_Ec_ /( b_Ec_ +10^(f+C)^)) • (r_f_• (100/(100+6^C^)) + r_f_mono_

Eq. 9: NH_4_^+^; ***d*A/*d*t** = Rp•μ_Rp_•R_A_•(1-(40/(40+1.29^N^))) - μ_Ec_•Ec/Y_A_ – (μ_Rp_•Rp/Y_AR_)•(A/(K_AR_+A))

Eq. 10: *E. coli*; ***d*Ec/*d*t** = μ_Ec_•Ec

Eq. 11: *R. palustris*; ***d*Rp/*d*t** = μ_Rp_•Rp

Eq. 12: Ethanol; ***d*e/*d*t** = Ec•3•(μ_Ec_•R_e_ + (G/(K_G_+G))•(10/(10+1.09^(1000•μEc)^))

• (b_Ec_/(b_Ec_+10^(f+C)^))•(r_e_•(100/(100+6^C^)) + r_e_mono_))

Eq. 13: CO_2_; ***d*CO2/*d*t** = Ec•6•(μ_Ec_•R_CO2_ + (G/(K_G_+G))•(10/(10+1.09^(1000•μEc)^))

• (b_Ec_/(b_Ec_+10^(f+C)^))•(r_co2_•(100/(100+6^C^)) + r_co2_mono_))

+ Rp•0.5•(μ_Rp_•Rh_Rp_ + r_Hp_•(C/(K_C_+C))•(40/(40+1.29^N^))•(b_Rp_/(b_Rp_+10^(f+C)^)))

Eq. 14: H_2_; ***d*H/*d*t** = Rp•(μ_Rp_•R_HRp_ + r_Hp_•(C/(K_C_+C))•(40/(40+1.29^N^)) •

(b_Rp_/(b_Rp_+10^(f+C)^))) + Ec•(μ_Ec_•R_HEc_ + (G/(K_G_+G))•(10/(10+1.09^(1000•μEc)^)) • (b_Ec_/(b_Ec_+10^(f+C)^))•(r_H_•(100/(100+6^C^)) + r_H_mono_))

Where,

μ is the specific growth rate of the indicated species (h^-1^).

μ_MAX_ is the maximum specific growth rate of the indicated species (h^-1^).

G, A, C, N, f, e, H and CO2 are the concentrations (mM) of glucose, NH_4_^+^, consumable organic acids, N_2_, formate, ethanol, H_2_, and CO_2_, respectively. All gasses are assumed to be fully dissolved. Consumable organic acids are those that *R. palustris* can consume, namely, lactate (3 carbons), acetate (2 carbons), and succinate (4 carbons). All consumable organic acids were simulated to have three carbons for convenience. Only net accumulation of formate, ethanol, CO_2_ and H_2_ are described in accordance with observed trends.

K is the half saturation constant for the indicated substrate (mM).

Ec and Rp are the cell densities (cells/ml) of *E. coli* and *R. palustris*, respectively.

b is the ability of a species to resist the inhibiting effects of acid (mM).

Y is the *E. coli* or *R. palustris* cell yield from the indicated substrate (cells / μmol glucose). Y values were determined in MDC with the indicated substrate as the limiting nutrient.

R is the fraction of glucose converted into the indicated compound per *E. coli* cell during growth (μmol of glucose / *E. coli* cell), except for R_A_. Values were adjusted to accurately simulate product yields measured in cocultures and in MDC with and without added NH_4_Cl.

R_A_ is the ratio of NH_4_^+^ produced per *R. palustris* cell during growth (μmol / *R. palustris* cell). The default value was based on that which accurately simulated empirical trends.

r is the growth-independent rate of glucose converted into the indicated compound (μmol / cell / h). Default values are based on those which accurately simulated empirical trends in coculture.

r__mono_ is the growth-independent rate of glucose converted into the indicated compound by *E. coli* when consumable organic acids accumulate. Default values are based on linear regression of products accumulated over time in nitrogen-free cell suspensions of *E. coli.*
