# Supplementary figures and images for: Recipient-Biased Competition for an Intracellularly Generated Cross-Fed Nutrient Is Required for Coexistence of Microbial Mutualists
Source: mBio. 2017 Nov 28;8(6):e01620-17. doi: 10.1128/mBio.01620-17 (PMC5705916; doi:10.1128/mBio.01620-17)

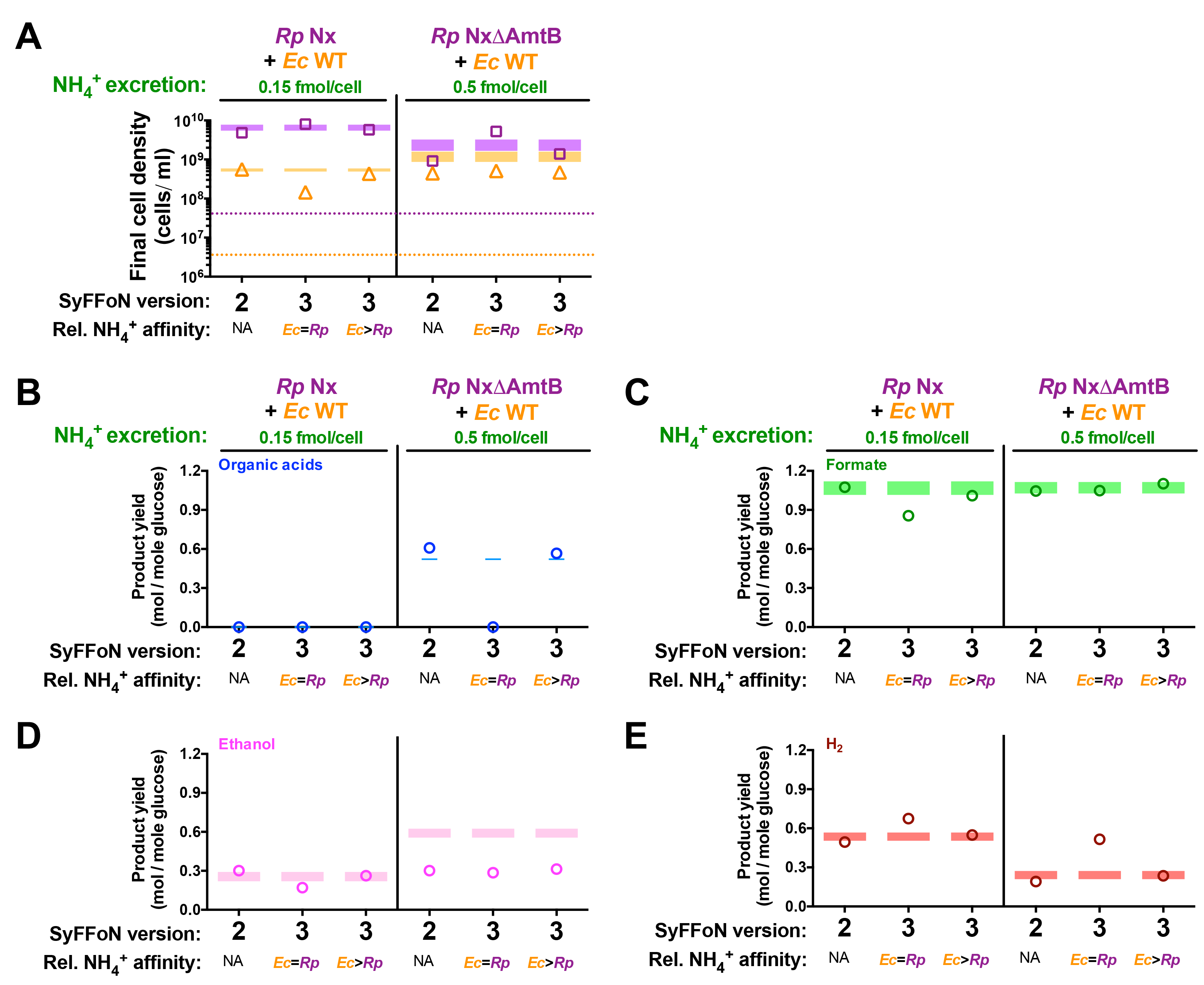

Supplement: FIG S1 [file mbo006173615sf1.tif]

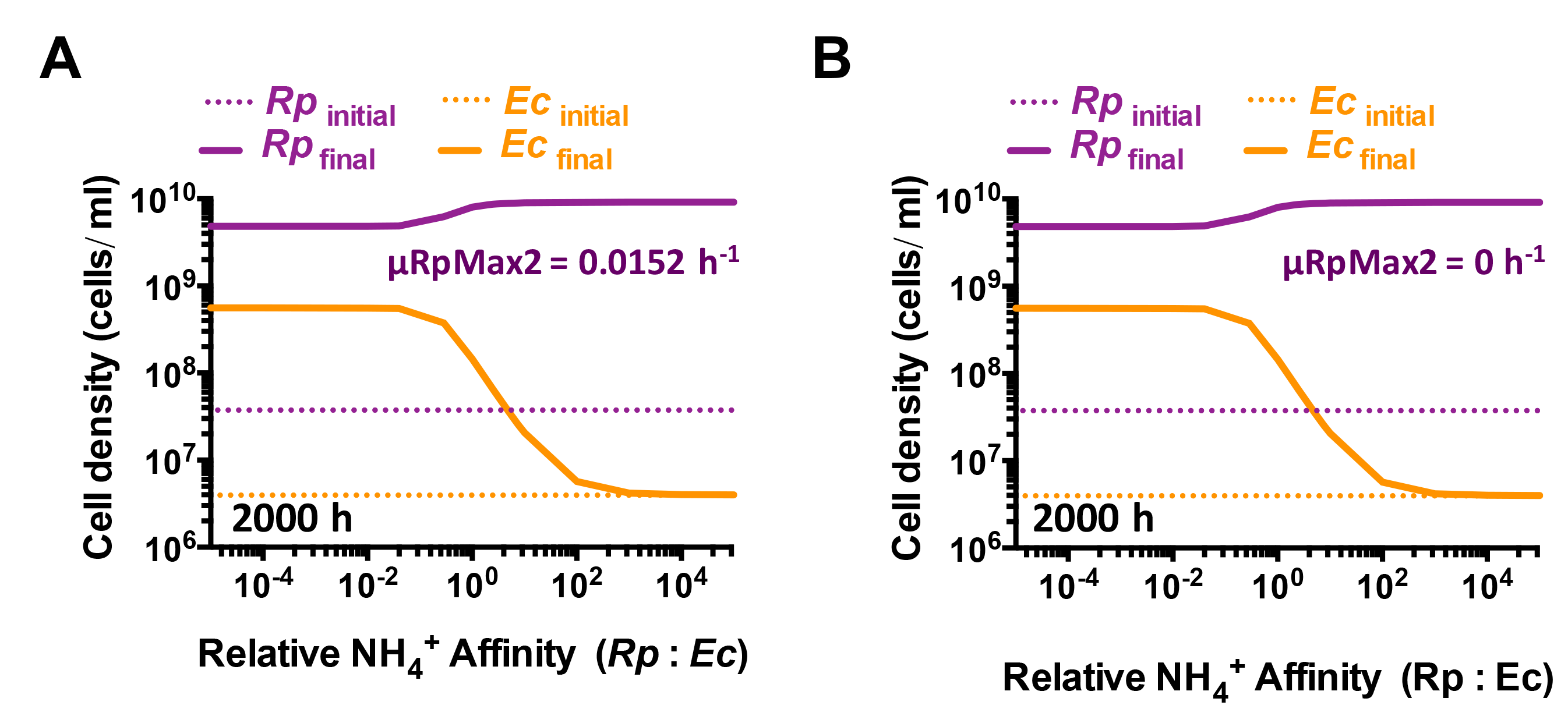

Supplement: FIG S2 [file mbo006173615sf2.tif]

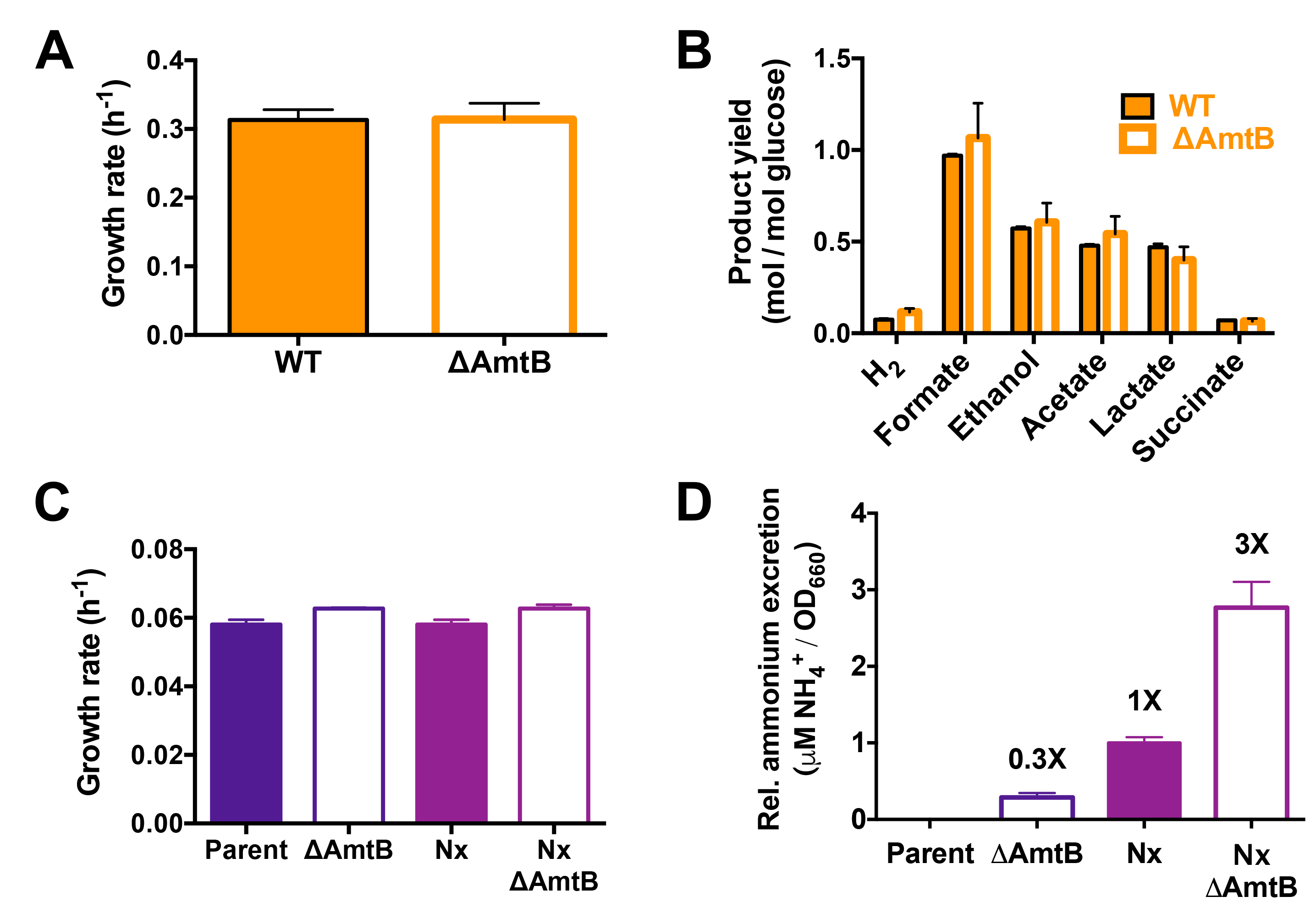

Supplement: FIG S3 [file mbo006173615sf3.tif]

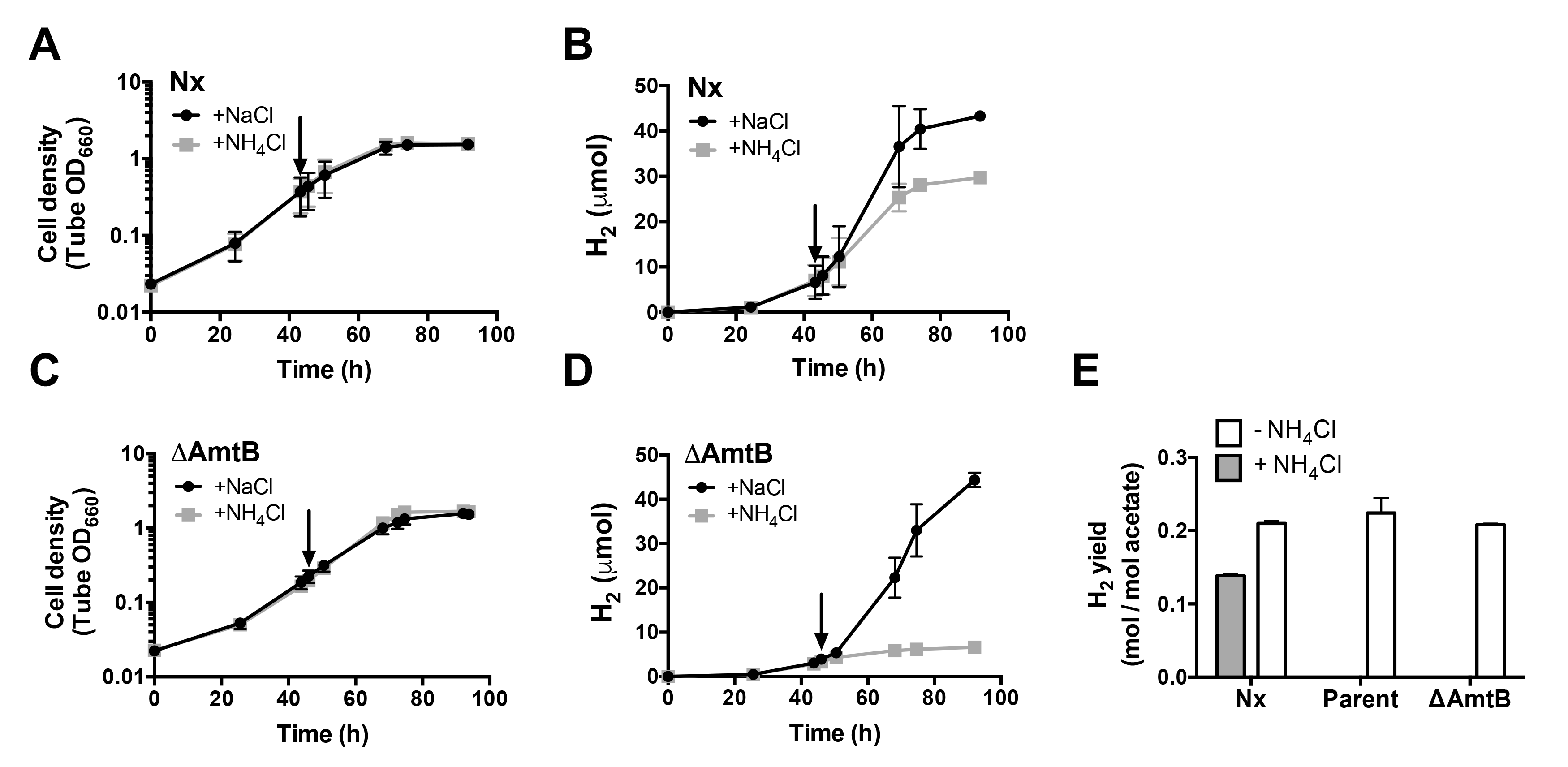

Supplement: FIG S4 [file mbo006173615sf4.tif]

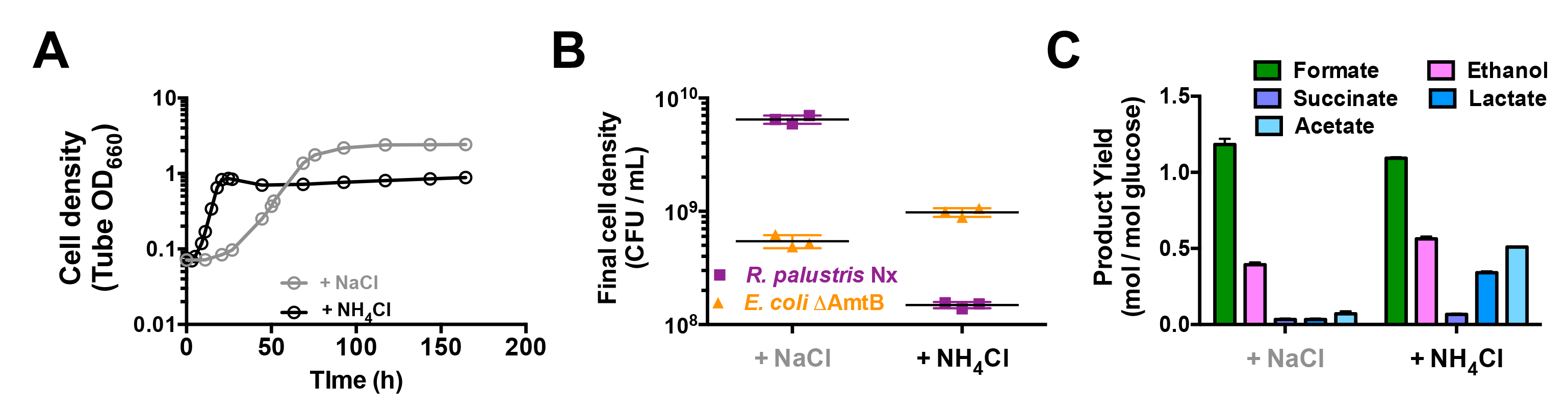

Supplement: FIG S5 [file mbo006173615sf5.tif]

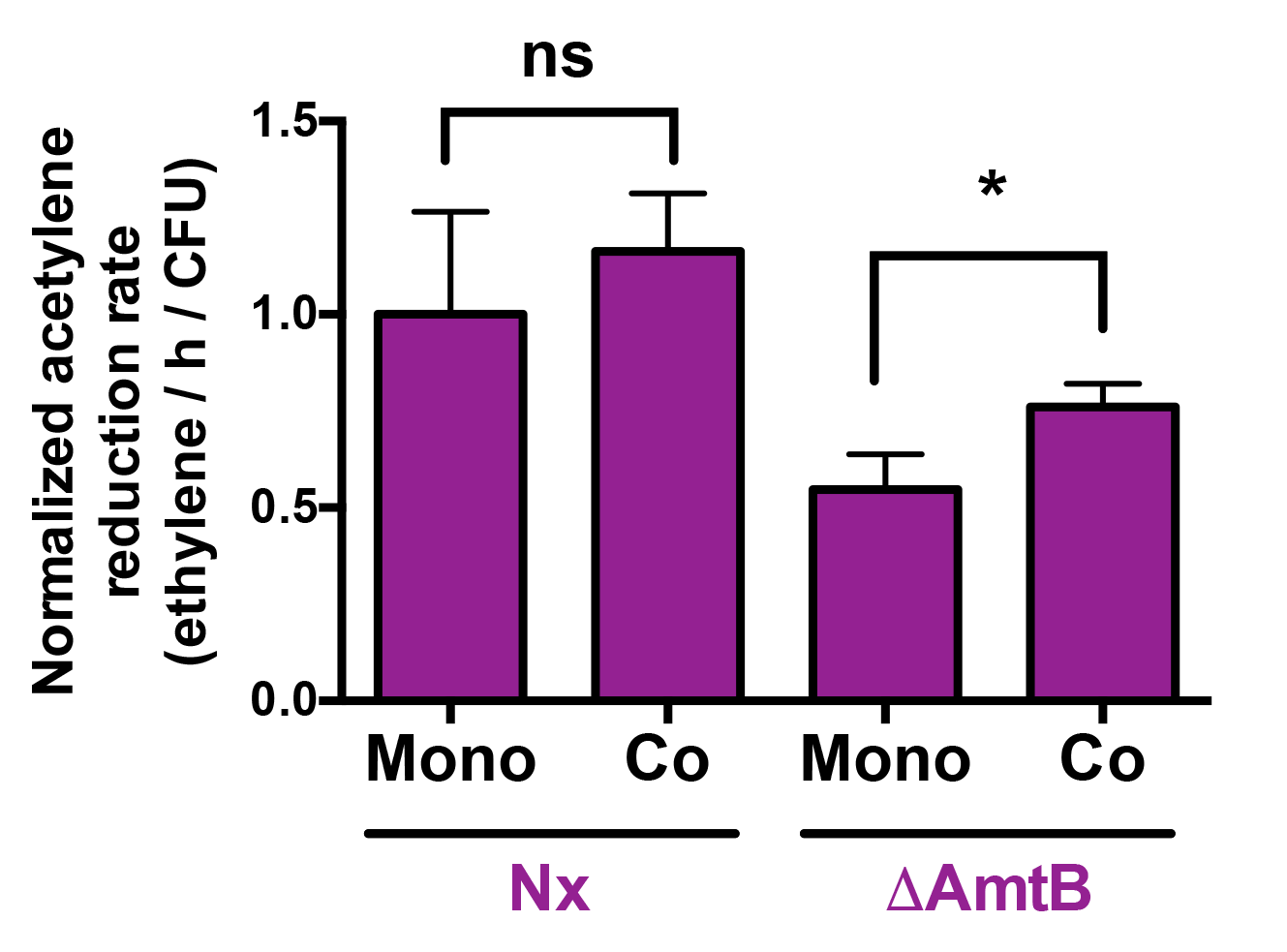

Supplement: FIG S6 [file mbo006173615sf6.tif]

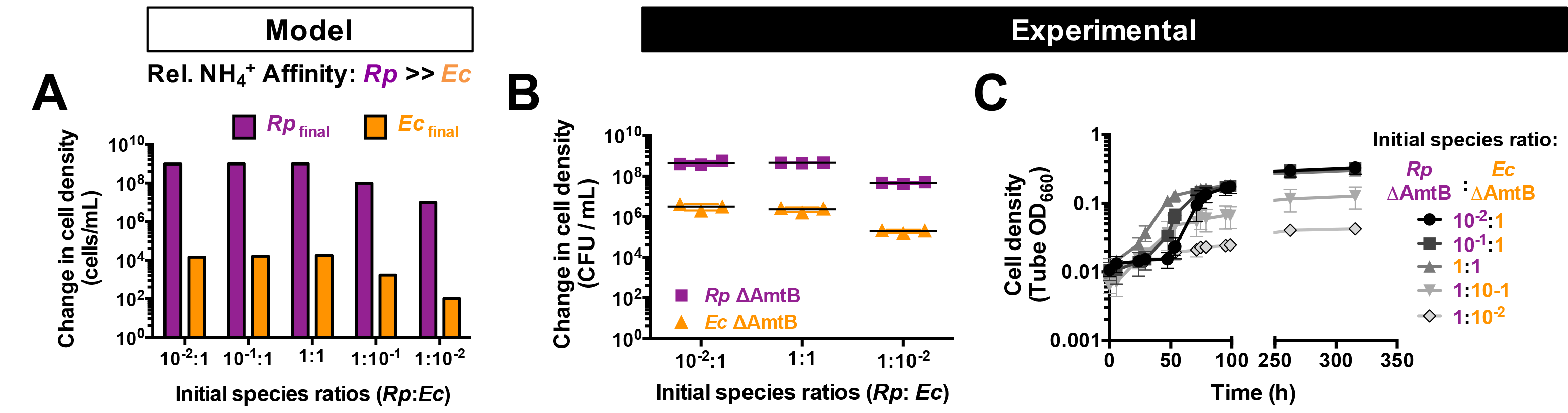

Supplement: FIG S7 [file mbo006173615sf7.tif]
