# Supplementary material for: Recipient-Biased Competition for an Intracellularly Generated Cross-Fed Nutrient Is Required for Coexistence of Microbial Mutualists
Source: mBio. 2017 Nov 28;8(6):e01620-17. doi: 10.1128/mBio.01620-17 (PMC5705916; doi:10.1128/mBio.01620-17)
Supplement: TABLE S2 [file mbo006173615st2.docx]

| **Strain or plasmid** | **Description or Sequence (5’-3’);**  **Designation** | **Source or Purpose** |
| --- | --- | --- |
| ***R. palustris* strains** | | |
| CGA009 | Wild-type strain; spontaneous Cm^R^ derivative of CGA001 | (1) |
| CGA4004 | CGA009 Δ*hupS* Δ*rpa2750*; Parent | (2) |
| CGA4005 | CGA4004 *nifA**; Nx | (2) |
| CGA4021 | CGA4005 Δ*amtB1* Δ*amtB2*; NxΔAmtB | (2) |
| CGA4026 | CGA4004 Δ*amtB1* Δ*amtB2;* ΔAmtB | This study |
| ***E. coli* strains** | | |
| MG1655 | Wild-type K12 strain, WT | (3) |
| K-12 JW0441-1 | Keio collection Δ*amtB::Km* | (4) |
| MG1655ΔAmtB | MG1655 Δ*amtB::Km*; ΔAmtB | This study |
| **Plasmids** | | |
| pJQnifA16 | Gm^R^; WT *nifA* gene flanked by XbaI/BamHI cloned into pJQ200SK | This study |
| **Primers** | | |
| \| ALM6f \| \| --- \| | TTCGTCGCTGAATTGCAACG | *amtB* upstream flanking region (*E. coli*) |
| ALM6r | TCAGGAAGGGGTGATGCGTA | *amtB* downstream flanking region (*E. coli*) |
| JBM1 | CGTctAgaccggcgcatcgc | *nifA16* upstream primer; XbaI |
| JBM6 | GGGGAtcctggttcgcagagg | *nifA16* downstream primer; BamHI |

Table S2 references.

1. Larimer FW, Chain P, Hauser L, Lamerdin J, Malfatti S, Do L, Land ML, Pelletier D a, Beatty JT, Lang AS, Tabita FR, Gibson JL, Hanson TE, Bobst C, Torres JLTY, Peres C, Harrison FH, Gibson J, Harwood CS. 2004. Complete genome sequence of the metabolically versatile photosynthetic bacterium *Rhodopseudomonas palustris*. Nat. Biotechnol. 22:55–61.

2. LaSarre B, McCully AL, Lennon JT, McKinlay JB. 2017. Microbial mutualism dynamics governed by dose-dependent toxicity of cross-fed nutrients. ISME J 11:337–348.

3. Hayashi K, Morooka N, Yamamoto Y, Fujita K, Isono K, Choi S, Ohtsubo E, Baba T, Wanner BL, Mori H, Horiuchi T. 2006. Highly accurate genome sequences of *Escherichia coli* K-12 strains MG1655 and W3110. Mol. Syst. Biol. 2:2006.0007.

4. Baba T, Ara T, Hasegawa M, Takai Y, Okumura Y, Baba M, Datsenko K a, Tomita M, Wanner BL, Mori H. 2006. Construction of *Escherichia coli* K-12 in-frame, single-gene knockout mutants: the Keio collection. Mol. Syst. Biol. 2:2006.0008.
